# To: Therapeutic hypothermia after cardiac arrest: outcome predictors

**DOI:** 10.5935/0103-507X.20160038

**Published:** 2016

**Authors:** Marina Verçoza Viana, Tiago Antonio Tonietto

**Affiliations:** 1 Hospital de Clínicas de Porto Alegre - Porto Alegre (RS), Brazil; Hospital Nossa Senhora da Conceição - Porto Alegre (RS), Brazil.

**To the Editor**,

Determining the neurological prognosis of patients who have suffered cardiac arrest is extremely important because it allows the physician to inform the family about the life expectancy of their beloved relative, as well as to wisely plan for the allocation of available resources. We read with great interest the study performed by Leão et al., who assessed factors associated with worse neurological outcomes after cardiac arrest.^(1)^

The study conducted by Leão et al. showed the ability of hypoxic-ischemic injuries viewed using magnetic resonance imaging of the brain to predict, in 72 hours, the prognosis of cardiac arrest survivors who had undergone therapeutic hypothermia (odds ratio - OR 19.8; 95% confidence interval - 95%CI: 1.7 - 229.6).^(1)^ This result reinforces the recommendations by the American Heart Association in regard to magnetic resonance imaging of the brain for neurological prognosis evaluation after cardiac arrest.^(2)^

The time from the return of spontaneous circulation until the target temperature was reached was also associated with the neurological prognosis. Patients who reached the target temperature more quickly presented worse neurological outcomes. However, we would like to highlight some important issues. First, the authors do not describe the initial temperature of the patients before they underwent therapeutic hypothermia. In addition, although hypothermia may reduce coronary perfusion, the fact that patients with more severe neurological damage were less reactive to low temperatures may make this finding a marker of worse prognosis and not necessarily its cause. The authors, as well as the editorial,^(3)^ also state that such results corroborate the findings of the randomized clinical trial performed by Kim et al. However, it is necessary to emphasize that this study was conducted only in out-of-hospital cardiac arrest and that induction of pre-hospital hypothermia increased the time spent by the team on site, possibly delaying interventions, such as cardiac catheterization, and increasing the number of cardiac arrests during transport.^(4)^

It is also important to note that the study population was heterogeneous (e.g., multiple initial cardiac arrest rhythms, distinct causes, in-hospital and out-of-hospital settings). These issues may have influenced patient outcomes.

Moreover, the authors did not evaluate whether patients with findings suggestive of worse prognosis had any limitations with regard to treatment or withdrawal of support, leading to self-fulfilling prophecies.

Lastly, the editorial^(3)^ considers that the findings of Leão et al.^(1)^ corroborate the maintenance of a temperature close to 36ºC. However, it was not the aim of the study to evaluate the impact of temperature control on cardiac arrest survivors, as all patients underwent hypothermia (33ºC target). The comparison with the study by Nielsen et al., which showed no difference between normothermia (36ºC) and hypothermia (33ºC), also requires some consideration. In the Targeted Temperature Management (TTM) trial, approximately 90% of cardiac arrest were witnessed, and individuals present at the scene started cardio-pulmonary resuscitation (CPR) in nearly 70% of the cases. These characteristics contribute to better neurological outcomes and may reduce the impact of more rigorous temperature control.^(5)^ For this reason, the guidelines of the American Heart Association suggest a wide range, from 32 to 36ºC, so that the target temperature can be individualized according to each patient.^(2)^

Marina Verçoza Viana and Tiago Antonio Tonietto

Hospital de Clínicas de Porto Alegre - Porto Alegre (RS), Brazil;

Hospital Nossa Senhora da Conceição - Porto Alegre (RS), Brazil.
